# Exploring Gender Perspectives in Medical Education: Latent Semantic Analysis of Israeli First-Year Medical Students’ Reflections

**DOI:** 10.2196/78371

**Published:** 2025-08-29

**Authors:** Rola Khamisy-Farah, Raymond Farah, Haneen Jabaly-Habib, Yara Nakhleh Francis, Nicola Luigi Bragazzi

**Affiliations:** 1 Azrieli Faculty of Medicine Bar-Ilan University Safed Israel; 2 Clalit Health Services Akko Israel; 3 Department of Internal Medicine B Ziv Medical Center Safed Israel; 4 Ophthalmology Department Tzafon Medical Center Poria Israel; 5 Department of Obstetrics and Gynecology Galilee Medical Center Nahariya Israel; 6 Laboratory for Industrial and Applied Mathematics (LIAM) Department of Mathematics and Statistics York University Toronto, ON Canada; 7 Department of Computer Science, Data Science, and Information Technology Faculty of Natural and Applied Sciences Sol Plaatje University Kimberley South Africa

**Keywords:** gender medicine, medical education, latent semantic analysis, cultural competence, reflective writing, student narratives, diversity, curricular assessment

## Abstract

**Background:**

Gender is increasingly recognized as a crucial determinant of health and health care delivery. Integrating gender-sensitive content into medical education is essential for cultivating socially responsive, culturally competent, and clinically effective physicians of the future. However, limited research has examined how medical students conceptualize gender in clinical contexts, particularly through their own reflective narratives.

**Objective:**

This study explores the thematic landscape of gender-related perceptions among first-year medical students in Israel following a mandatory course in gender medicine. Using latent semantic analysis (LSA), we examined how students reflected on gendered dimensions of health care and how these reflections varied by gender and ethnicity.

**Methods:**

First-year medical students enrolled in the four-year path of medicine in Israel participated in a compulsory gender medicine course and were invited to submit anonymous written reflections. A total of 83 students (n=52, 63%, females; n=31, 37%, males; n=68, 82%, Jewish; and n=15, 18%, Arab) submitted responses, which were preprocessed and analyzed using LSA. The texts were lemmatized and vectorized to construct a term-document matrix, followed by singular value decomposition for dimensionality reduction. Ten latent topics were extracted, and thematic labels were assigned through an inductive, consensus-based coding procedure. Subgroup analyses were conducted by gender and ethnicity.

**Results:**

LSA identified 10 distinct topics, accounting for 56.6% of the total variance in the overall sample. The most dominant theme was Gendered Patient-Doctor Interactions (eigenvalue=121.188; 28.1% variance; 527 terms; 75 documents), followed, in terms of variance, by Gender-Specific Diseases and Health Concerns (5.7%) and Cultural and Religious Influences on Health Care (4.3%). Reflections from female students introduced 3 unique themes: Gendered Help-Seeking and Familial Roles (2.8%), Gender and Health Education (2.5%), and Gendered Communication and Advocacy (2.2%). Male students uniquely discussed Perceived Gender Bias in Clinical and Research Settings (3.8%) and the Legal and Ethical Dimensions of Reproductive Health Care (3.3%). Among Jewish students, additional themes included Population-Level Framing of Gendered Conditions (3.7%) and Gendered Youth Expectations (2.1%). Arabic students contributed culturally specific themes, such as Modesty and Cultural Norms (8.6%), Paternal Authority and Structural Discrimination (6.3%), and Reproductive Vulnerability (3.6%).

**Conclusions:**

Thematic patterns in student reflections suggest that gender medicine curricula are effective in fostering critical engagement with diverse gendered realities in clinical care. The emergence of culturally grounded and gender-specific themes underscores the importance of tailoring educational interventions to reflect student diversity.

## Introduction

Sex and gender are distinct yet interrelated factors that shape health, health care access, and, ultimately, health outcomes in multiple and profound ways [1]. While sex refers to biological attributes such as chromosomes, hormones, and reproductive anatomy that typically categorize individuals as male or female, gender encompasses the socially constructed roles, behaviors, expressions, identities, and power relationships associated with being a man, woman, or gender-diverse person [2,3]. As a social determinant of health, gender intersects with biological factors as well as with other cultural, societal, and systemic constructs to influence the incidence, prevalence, and manifestation of diseases, diagnostic pathways, therapeutic decisions, clinical interactions, and patient experiences across the care *continuum* [4-6].

Despite increasing recognition of these dynamics and interwoven interplays, medical education has historically followed a reductionist biomedical model that often underrepresents or insufficiently integrates sex- and gender-sensitive perspectives [7]. In recent years, sex- and gender-based medicine has emerged as a multidisciplinary field aimed at addressing these gaps by incorporating the complexities of sex- and gender-specific differences into clinical education, research, and practice [8].

Embedding sex- and gender-based medicine into undergraduate medical curricula is essential for preparing future physicians to recognize and respond to sex- and gender-based disparities in health care delivery, enhance diagnostic accuracy, and foster more equitable patient care [9,10]. However, most medical schools do not offer a formal, integrated curriculum in sex- and gender-sensitive medicine; when included, content is often confined to reproductive health and rarely extends to other specialties such as cardiology, pharmacology, or psychiatry. Educators have expressed concerns about the lack of standardized materials, institutional support, and faculty preparedness, all of which hinder implementation. Students, while generally receptive, often report minimal exposure to these topics, and when presented, content is frequently framed in binary, biologically deterministic terms [11]. Despite these limitations, increasing evidence shows that structured educational interventions can raise awareness of implicit bias and promote more equitable clinical practice [12,13].

Yet little is known about how students internalize and reflect on sex- and gender-sensitive content when it is presented within a formal curriculum [14]. This gap underscores the need for qualitative and data-driven evaluations of student perspectives to better inform curriculum design and pedagogical strategies. Narrative reflection provides a window into students’ evolving attitudes and cognitive engagement with sex and gender in clinical contexts [14,15]. It is a tool grounded in constructivist learning theory, which posits that learners actively construct knowledge through experience, reflection, and social interaction rather than passively absorbing facts [15]. Rooted in the work of Piaget, Vygotsky, and others, constructivism emphasizes that meaning-making is contextual and shaped by prior knowledge, cultural frameworks, and active engagement. In health professions education, constructivist approaches have gained traction as educators recognize the limitations of didactic instruction in preparing students for the complex, value-laden, and relational aspects of clinical care, such as those encountered in sex- and gender-based medicine. Reflective writing further enables learners to make sense of the social and ethical dimensions of medicine, including issues of power, identity, and equity [16].

By leveraging narrative reflection and a computational linguistic framework, this study aims to elucidate how students conceptualize sex and gender in medical practice and to identify thematic patterns that vary by gender and ethnicity within a diverse multicultural educational context. Through written reflections, students engage in metacognitive processing of sex- and gender-related content presented during the course. These narratives serve not only as evidence of individual meaning-making but also as artifacts that reveal the evolving cognitive, emotional, and ethical dimensions of learners’ understanding. Within the constructivist paradigm, reflection functions as both a tool and a product of learning, enabling students to surface biases, grapple with complexity, and reconcile new information with existing worldviews.

Specifically, we asked (1) what thematic patterns emerge in first-year medical students’ reflections following a formal course in sex- and gender-based medicine? and (2) how do these patterns differ by students’ gender and ethnicity? Based on prior literature on sex and gender awareness in medical education [12,13,17,18], we hypothesized that the reflections would reveal themes capturing both clinical and sociocultural dimensions of sex and gender in medicine. These themes were expected to include recognition of sex- and gender-specific differences in disease incidence, prevalence, presentation, and outcomes; awareness of gender bias in clinical practice as presented during the course; and acknowledgment of the role of sociocultural norms in shaping health care experiences. Given the absence of prior extensive clinical exposure, we anticipated that students’ narratives would primarily integrate knowledge gained from the structured curriculum with their preexisting personal, cultural, and societal perspectives on sex, gender, and health. We further expected thematic emphases to vary by students’ gender, with potential differences in the extent to which narratives addressed interpersonal, affective, and patient-centered aspects of care alongside structural, biomedical, or systems-level considerations. We also anticipated that students from different ethnic backgrounds would foreground culturally specific norms, values, and challenges in applying sex- and gender-based medicine principles. By articulating these differences, this study aims to provide medical educators with evidence to design more culturally responsive and pedagogically effective curricula in sex- and gender-sensitive medicine.

## Methods

### Study Population

The participants were first-year medical students enrolled in the 4-year medical program at the Azrieli Faculty of Medicine, Bar-Ilan University, Safed, Israel. They attended a mandatory sex- and gender-based medicine course as part of their curriculum. All students provided written reflections on their experiences and learning outcomes, which constituted the textual dataset for analysis.

### Ethical Considerations

This study adhered to ethical guidelines for educational research. Written informed consent was obtained from all students before data collection. Institutional ethical approval was granted by the Human Subjects Institutional Review Board at Bar-Ilan University, Safed, Israel (approval number 040325496). Participation was entirely voluntary, and students’ reflections were anonymized and used solely for academic and research purposes.

### Course Syllabus

The course Sex- and Gender-Based Medicine was designed to introduce and deepen understanding of key concepts, theories, and practices in sex- and gender-sensitive medicine, emphasizing how sociocultural and institutional determinants shape health disparities.

Over 7 structured first-year sessions, each consisting of a 90-minute plenary lecture followed by a 90-minute small-group practice, students developed both conceptual and applied competencies for integrating a sex- and gender-aware perspective into clinical practice and medical research.

Using Bloom’s taxonomy [19], the learning progression moved from remembering (defining sex and gender; describing biological, social, and cultural determinants) and understanding (explaining how sex and gender influence biological systems, disease manifestation, health-seeking behavior, and access to care) to applying (using a sex- and gender-sensitive lens to analyze case studies such as women with chest pain or men with breast cancer), analyzing (identifying bias in biomedical research design and interpretation), evaluating (assessing the impact of policy, feminist theories, and global perspectives on health care delivery), and creating (formulating strategies to address inequities and adapt medical innovations for diverse populations).

Learning activities combined plenary lectures with small-group sessions featuring case-based discussions, simulations with standardized patients, podcast analyses, and reflective exercises. The curriculum integrated national and global perspectives, traced the historical evolution of feminist thought and its influence on medicine, examined gender gaps in research, and critically analyzed the effects of bias on health care delivery and innovation.

Students were required to attend at least 80% of sessions and submit a written reflection, for instance, applying a sex- and gender-sensitive framework to a clinical scenario, thereby demonstrating mastery of course objectives across multiple levels of cognitive engagement.

### Reflective Writing Assignment and Prompts

At the conclusion of the Sex- and Gender-Based Medicine course, students were required to submit a 1-page written reflection as a mandatory graded assignment. The prompt invited them to describe and critically analyze their learning experience, drawing on lectures, small-group discussions, case-based exercises, or personal experiences relevant to sex- and gender-sensitive medicine. Students were encouraged to focus either on a specific event or on their overall experience, integrating description, interpretation, and self-analysis.

The assignment was structured around the DIEP (Describe, Interpret, Evaluate, and Plan) model guiding students to (1) describe the chosen experience or case; (2) interpret the actions, motives, emotions, and contextual factors involved; (3) evaluate their own responses and the quality of their engagement; and (4) plan how to apply the insights gained to future academic, clinical, or research contexts. To support the reflective process, students were given stepwise guidance: identifying the experience, describing the learning process, engaging in introspection, recognizing shifts in perspective or values, and directing the reflection toward future practice. They were encouraged to write in the first person and connect their reflections to personal perspectives and professional aspirations. Model phrases were provided to help articulate reflective thinking (eg, “This experience made me aware of...”, “Looking back at what happened...”). All submissions received individualized feedback aimed at deepening reflective skills.

### Data Collection and Preprocessing

The dataset consisted of written responses submitted by students at the conclusion of the course. These reflections were collected electronically and compiled into a structured corpus for text mining. The responses varied in length and complexity, reflecting diverse perspectives on gender-related topics in medicine. Before analysis, all textual data underwent preprocessing to ensure the reliability of results. This included tokenization, stop-word removal, stemming, and lemmatization. Text was converted to lowercase, and punctuation and nonalphabetical characters were removed to standardize the dataset. Common medical terminology and gender-related terms were retained to preserve contextual relevance. Preprocessing was performed using the English stopword list and English-language stemming algorithms.

### Statistical Analysis

This study used latent semantic analysis (LSA) to examine the perceptions and conceptualizations of gender medicine among Israeli first-year medical students. LSA is a computational information-processing method for humanlike, meaning-based cognitive tasks, including the extraction of conceptual meaning from large textual *corpora*. It addresses the question of “how word and passage meaning can be constructed from experience with language, that is, by what mechanisms—instinctive, learned, or both—this can be accomplished” [20]. In LSA, “words do not have meanings on their own..., words get their meanings from their mapping” [20], and it is “the underlying map (that) is (to say) the primitive substrate that gives words meaning, not vice versa” [20]. LSA recognizes that people rarely communicate using isolated words but typically speak and comprehend clusters of words, often extending beyond a single sentence. Such units of meaning are more accurately represented at the level of paragraph-length discourse.

More technically speaking, LSA relies on the “compositional constraint,” which posits that “the representation of any meaningful passage must be composed as a function of the representations of the words it contains” [20]:



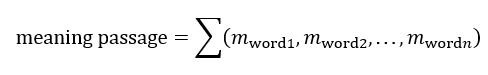



“LSA learns about the meaning of a word from every meeting with it and from the composition of all the passages in which it does not occur...Word meaning is latent in the evidence of experience and can be extracted from natural linguistic data” [20].

The LSA technique has been increasingly applied in health professions education to analyze reflective narratives and explore students’ mental models [21,22]. By identifying patterns of word co-occurrence and reducing linguistic dimensionality, LSA detects latent themes that may not be readily apparent through manual coding alone. It thus offers a scalable, data-driven approach to evaluating the impact of educational interventions, particularly in areas involving subjective and contextually embedded content such as gender.

A term-document matrix was constructed using a bag-of-words representation with a minimum term frequency of 2 and a sparsity threshold of 0.975. Singular value decomposition was applied to reduce dimensionality and reveal key semantic relationships between words. The number of topics was determined using an optimal singular value selection approach to capture the most relevant themes without overfitting the data. LSA was first performed on the overall sample to identify global themes and semantic structures, followed by stratified analyses by gender and ethnicity.

Following LSA, the resulting topics were manually reviewed and labeled according to their most representative words. Two independent researchers (RKF and NLB) conducted the topic interpretation to ensure reliability, resolving discrepancies through discussion until consensus was reached. A hybrid coding approach was applied: an initial a priori codebook was developed based on the study’s research questions and prior literature on sex- and gender-based medicine. This preliminary framework included categories expected to capture both clinical and sociocultural dimensions of student reflections. The codebook was then iteratively refined in a data-driven manner, consistent with constructivist theory, through manual review of the topic-modeling outputs and a subset of student narratives. This process allowed for the addition of new codes and the consolidation or clarification of existing ones. In doing so, the final set of codes reflected both theoretically informed expectations and emergent themes in the data, thereby enhancing the validity and reproducibility of the analysis.

The codebook, including a priori categories, their definitions, and emergent subcodes within each category, is presented in Table 1.

All LSA procedures were conducted using XLSTAT (Lumivero, LLC).

**Table 1:** Codebook for the thematic analysis.

A priori category	Definition	Emergent subcodes
Clinical Applications of Sex- and Gender-Based Medicine	Recognition of how sex and gender influence disease incidence and prevalence, presentation, outcomes, pharmacology, and decision-making in clinical contexts	Gender and Decision-Making in Medicine; Gender Differences in Pharmacology; Gender Differences in Pharmacology and Disease Education; Gender-Specific Diseases and Health Concerns; Reproductive Health and Feelings of Vulnerability
Sociocultural Influences on Health	Influence of cultural, religious, and social norms on health beliefs, access, and care delivery	Cultural and Religious Influences on Health Care; Cultural and Social Attitudes Toward Gender and Health; Cultural Norms in Clinical Settings; Gendered Help-Seeking and Familial Roles; Gendered Youth, Expectations, and Identity Formation; Intergenerational and Familial Perspectives on Gendered Health
Educational and Institutional Contexts	The role of medical education, institutional structures, and team dynamics in shaping awareness and practice of gender-sensitive medicine	Gender and Health Education; Gender Equality and Team Dynamics; Gender Roles in the Medical Profession; Institutional and Interpersonal Perspectives on Gender; Perceived Gender Bias in Clinical and Research Settings
Health Access and Equity Issues	Barriers and facilitators to equitable health care for diverse gender identities	Gender and Public Health Framing of Conditions; Health Care Access for Transgender Individuals; Legal and Ethical Dimensions of Gendered Health Care
Gendered Communication and Relationships	How gender influences patient-provider interactions, communication styles, and relational dynamics in care	Gendered Clinical Encounters; Gendered Communication and Assertion in Health Care Contexts; Gendered Patient-Doctor Interactions
Psychological and Emotional Dimensions of Gender and Health	Emotional, mental health, and psychosocial aspects linked to gender identity or experience	Paternal Authority and Structural Discrimination; Psychological and Emotional Dimensions of Gender and Health; Psychosocial Support and Gendered Distress

## Results

### Overview

All 83 students were recruited. Of these, 52 (63%) identified as female and 31 (37%) as male. In terms of ethnicity, 68 participants (82%) identified as Jewish and 15 (18%) as Arab.

In the overall sample analysis, LSA (Table 2) revealed a rich and multidimensional thematic structure, highlighting the broad spectrum of gender-related issues perceived by students in the context of medical education and clinical care.

In total, 10 distinct topics were identified. Topic 1 exhibited the highest eigenvalue (121.188), accounting for approximately 28.1% of the total variance, and demonstrated high lexical richness (527 terms) as well as strong document representation (75 documents). The primary terms within this topic indicate that students engaged critically with gendered dynamics in patient-doctor interactions, emphasizing gender as a determinant of health and its implications for clinical communication, empathy, and diagnostic equity. Beyond this dominant theme, topic 2, with an eigenvalue of 54.519 (5.68% of the explained variance), reflected students’ awareness of gender-specific health concerns, particularly those disproportionately affecting women. This thematic axis reflects recognition of both the biological and social dimensions of illnesses such as breast cancer and their ramifications for patient care and support systems. Topic 3, with an eigenvalue of 47.175 and accounting for 4.26% of the overall variance, indicates that students considered how religious beliefs and cultural contexts intersect with gender identity and health care experiences, particularly for conditions such as endometriosis that are not only underdiagnosed but also socially stigmatized. This highlights the students’ developing sensitivity to culturally competent care and the complexities of treating individuals within diverse socioreligious frameworks. The subsequent topics (topics 4-10) each accounted for between 3.49% and 1.90% of the variance, with eigenvalues ranging from 42.725 to 31.507. Topic 4 addressed the complex role of gender in shaping professional identities and hierarchies in medicine. This theme was further reflected in topics 7 and 8, which collectively examined gender-based dynamics in medical decision-making, team interactions, and perceived equality in clinical and academic settings. Topic 7 highlighted heightened student awareness of the gendered structures influencing teamwork, authority, and career progression within medicine, while topic 5 underscored students’ growing recognition of transgender health concerns and the structural barriers to inclusive health care delivery. Topic 6 focused on gender pharmacology—particularly the often underappreciated physiological and hormonal differences that affect drug efficacy and therapeutic response. Topic 9 addressed students’ reflections on interpersonal and institutional factors shaping gendered experiences within medical learning environments. Finally, topic 10 conveyed a nuanced consideration of the psychological and normative dimensions of gendered illness narratives, emphasizing students’ awareness of how gender norms shape both the perception and lived experience of illness, as well as the societal frameworks governing the provision of emotional support and care.

**Table 2:** Labeled topics identified through latent semantic analysis of student reflections following participation in a gender medicine course. Each topic is characterized by its corresponding eigenvalue, percentage of explained variability, key terms, and the thematically assigned label.

Topic	Label	Representative quotes	Eigenvalue	% Variability	Top words
Topic 1	Gendered Patient-Doctor Interactions	“For me, this was the first time I had to deal with questions of gender identity and the complex feelings of her and the team...” “. I intend to avoid pre-assumptions about gender identity and pay attention to how the environment may affect how patients feel...”	121.188	28.08	gender, patient, women, feel, doctor
Topic 2	Gender-Specific Diseases and Health Concerns	“We learned how much awareness is lacking about the disease among men, how male patients with breast cancer have to cope with coming to a ward where all the patients are women, and how their image is affected because the disease is perceived as a women's disease...” “In our world, the word “breast” or “breasts” is perceived as a feminine and maternal organ (for feeding a baby), and telling a man that he has breasts can be very difficult for him...”	54.519	5.68	diseas, breast, cancer, partner, didn
Topic 3	Cultural and Religious Influences on Health Care	“I spoke to my grandparents and tried to explain the situation to them in simple words, without medical terminology, I clarified that it would be documented in her medical record and that there would be no societal reason to look down on her, I reminded them that preventing treatment and causing harm to a person contradicts our religion...” “One particular issue that deeply resonated with me that was raised during the course is the prolonged time it often takes to diagnose endometriosis in women...”	47.175	4.26	peopl, comfort, religi, comprehens, endometriosi
Topic 4	Gender Roles in the Medical Profession	“I thought to myself why I was actually hurt by his behavior, maybe because I wanted to prove that I could fulfill the role no less than a male paramedic and I wanted to receive the treatment I deserved as a team leader, as a woman, as someone who spoke a different language...” “Dr. *** described to us how she participated in a discussion during one of the meetings of the heads of department, she was one of the few women, if not the only woman in such a senior and managerial role, surrounded by male colleagues...”	42.725	3.49	complex, role, profess, realiti, sick
Topic 5	Health Care Access for Transgender Individuals	“In our work as doctors, we may be required to address the unique issues of the transgender population...” “The case I chose to describe occurred several years ago and concerns my own and society’s attitude toward a transgender soldier...”	40.302	3.11	base, transgend, servic, unit, serv
Topic 6	Gender Differences in Pharmacology	“I knew that drugs could affect women and men differently, but I was surprised to discover the extent of the difference...” “We were told that most drugs have been and are still being studied in men only...”	38.321	2.81	lectur, drug, dose, menstrual, gap
Topic 7	Gender and Decision-Making in Medicine	“This exposure pushed me to reflect on how gender-associated stigma influences my own life and decision-making...” “Returning to what happened in this case, it is clear that unconscious gender biases influenced medical decisions...”	36.769	2.59	student, woman, decis, separ, stori
Topic 8	Gender Equality and Team Dynamics	“I had always treated all the women around me with respect and it was clear to me that men and women were equal...” “My values, based on human dignity and equality, pushed me to act in the girl's best interest...”	35.082	2.35	sister, equal, team, dr, incid
Topic 9	Institutional and Interpersonal Perspectives on Gender	“I feel that I have received significant tools for dealing with questions from patients, friends, and acquaintances on various topics...” “In addition, I learned from the case the importance and need for raising awareness of the issue and the great importance of training the staff to respect the identity of each person and allow them to receive the best medical care for them...”	34.349	2.26	subject, friend, biolog, staff, liber
Topic 10	Psychological and Emotional Dimensions of Gender and Health	“It was important to me that, during the meeting, she express herself to the doctor and raise the points that concerned her, so she could feel comfortable and overcome her fear and embarrassment...” “My choice of a gynecologist (rather than a doctor) was made mainly due to discomfort or fear of being examined by a male doctor, following unpleasant experiences and situations experienced by my friends and family...”	31.507	1.90	ill, fear, syndrom, norm, save

### Subanalysis by Gender

The LSA of reflections from female medical students (Table 3) revealed that while several key topics were retained from the full-sample analysis, new themes also emerged, and some existing ones underwent lexical refinement and thematic expansion.

Topic 1 remained the most dominant, with topic 2 also consistent. Topic 3, however, demonstrated a partial thematic reorientation: while previously aligned with cultural and religious dimensions, it now emphasized communication and generational perceptions over doctrinal belief. Topic 4 incorporated a broader intersectional framing of gender complexity and patient autonomy. Similarly, topic 5 integrated educational and clinical discourse, with a particular focus on conditions such as breast cancer. Distinctively, several new themes emerged uniquely within the female-only dataset. Topic 6 introduced a previously unarticulated theme of Gendered Help-Seeking and Familial Roles, possibly reflecting caregiving norms and informal consultation practices. Topic 7 clarified the psychological and emotional dimensions of gender and health. Topic 8 suggested a pedagogical and developmental framing, while topic 9 reflected an intergenerational perspective on gendered health experiences. Finally, topic 10 revealed a discourse on self-expression, advocacy, and possible resistance to gendered dismissal or invalidation.

The LSA of reflections from male medical students (Table 4) showed that while most identified topics were retained, some exhibited lexical drift and partial thematic reorientation.

For instance, topic 6 indicated a shift toward a more biologically grounded understanding of gender. Topic 8 appeared to merge interpersonal reflections with the emotional and physical dimensions of team-based care, thereby becoming lexically broader in scope. Two new themes also emerged in the male-only analysis. Topic 9 reflected critical engagement with perceived gender bias in diagnostic processes and biomedical research. Finally, topic 10 introduced a novel legal and ethical discourse on reproductive health, suggesting that at least some male students approached gendered issues through a rights-based and normative framework.

**Table 3:** Labeled topics identified through latent semantic analysis of student reflections following participation in a gender medicine course in the female-only sample.

Topic	Label	Representative quotes	Eigenvalue	% Variability	Top words
Topic 1	Gendered Patient-Doctor Interactions	“The patient's actions and motivations stem from the enormous difficulty of dealing with rejection from her father and the lack of acceptance of the gender change she underwent...” “When I went to the doctors to find out the cause, they said it would pass, that I was a girl experiencing stress, or that it might be anxiety...”	101.038	31.89	patient, gender, doctor, women, medic
Topic 2	Gender-Specific Diseases and Health Concerns	“I learned a lot from my sister’s case, I knew that I wanted to be a doctor even before the experience we went through as a family, but the aforementioned experience sharpened for me the importance of seeing the patient as a whole...” “At that time, I had been with my partner for about a year, I asked him to accompany me to the gynecologist due to pain I was experiencing...”	44.608	6.22	diseas, examin, sister, gynecologist, hospit
Topic 3	Cultural and Social Attitudes Toward Gender and Health	“From this experience, I realized that due to my gender and the doctor's prejudices, I received treatment that, in my opinion, was improper and disparaging...” “I think that there is at least some kind of shame and discomfort in society to talk about the subject, especially for a girl and even more challenging in an environment with boys...”	39.634	4.91	girl, peopl, opinion, talk, convers
Topic 4	Health Care Access for Transgender Individuals	“During my work as a nurse in the emergency room, I treated a patient who identified as a transgender woman...” “The patient had a psychiatric background and a past suicidal experience, which stemmed from the insults and bullying she experienced for being a transgender woman...”	36.700	4.21	respect, complex, menstrual, transgend, oper
Topic 5	Gender Differences in Pharmacology and Disease Education	“For example, we talked about breast cancer, which in most cases only affects women, and that is why most of the health system focuses on women...” “I realized that the patient experienced side effects following the dosage of the drug and that gender differences that may affect the pharmacokinetics and pharmacodynamics of the drug cannot be ignored...”	33.003	3.40	breast, cancer, lectur, student, drug
Topic 6	Gendered Help-Seeking and Familial Roles	“A few months ago, my aunt had a general check-up with her gynecologist, the results showed that she had a large polyp on her cervix...” “I felt anger toward both society and my grandparents because delaying the treatment would put my aunt's health and life at risk...”	30.062	2.82	aunt, help, step, consult, recommend
Topic 7	Psychological and Emotional Dimensions of Gender and Health	“I assume that this was due to the emotional difficulties that the syndrome brings with it...” “The doctor did not delve into the impact of the pain on my moods and did not refer me to further advice, but only gave me a prescription...”	28.420	2.52	discuss, mention, syndrom, save, delv
Topic 8	Gender and Health Education	“I plan to...continuously educate myself on gender and sex-specific health issues to improve my diagnostic skills...” “Medical education that emphasizes sex and gender medicine may improve awareness of many specific diseases, especially endometriosis, and lead to improved diagnosis and treatment...”	28.052	2.46	period, member, educ, boy, reach
Topic 9	Intergenerational and Familial Perspectives on Gendered Health	“Being nervous didn't make the exam any easier, and my mother, in her response, increased my concerns...” “This interested me because my mother had breast cancer and underwent a partial mastectomy and no doctor in the process talked about this issue...”	27.066	2.29	didn, mother, comprehens, comfort, age
Topic 10	Gendered Communication and Assertion in Health Care Contexts	“I remember trying to explain to the person in charge that I believed in that girl's pain, and that I thought it was better to leave her to rest in the room than to force her to travel while she was suffering...” “I feel like I didn't handle this situation very well because I let my emotions, the feeling that I felt attacked, manage my way of talking...”	26.382	2.17	group, explain, respond, don’t, attack

**Table 4:** Labeled topics identified through latent semantic analysis of student reflections following participation in a gender medicine course in the male-only sample.

Topic	Label	Representative quotes	Eigenvalue	% Variability	Top words
Topic 1	Gendered Patient-Doctor Interactions	“It is possible that this was caused by unconscious gender stereotypes, as although the symptoms were typical of a heart attack, the doctors initially attributed them to stress or anxiety, delaying the correct diagnosis and appropriate treatment...” “In the course I had the experience of getting exposed to gender-based challenges in the health system...”	72.569	25.97	gender, feel, women, men, medic
Topic 2	Gender-Specific Diseases and Health Concerns	“During the Gender Medicine course we were presented with a scenario that highlighted the unique challenges faced by men who have been diagnosed with breast cancer, a condition primarily associated with women...” “For men dealing with diseases that are perceived as feminine, such as breast cancer, the lack of appropriate gender support can lead to feelings of not belonging and even avoidance of receiving essential care...”	41.085	8.32	breast, cancer, incid, –, moment
Topic 3	Health Care Access for Transgender Individuals	“The incident I chose to describe happened a few years ago and deals with the attitudes of myself and society towards a transgender soldier, the incident highlights challenges in military service...” “Indeed, the soldier arrived at my unit for a professional evaluation, while the HR personnel explained to me the complex living and service conditions that must be provided to a transgender soldier and how difficult it would be to arrange these conditions in my unit and therefore, they suggested it was not advisable for me to “deal” with such a soldier...”	39.584	7.73	base, transgend, servic, unit, train
Topic 4	Cultural and Religious Influences on Health Care	“This case highlighted the need to find a balance between respecting religious beliefs and maintaining equality and inclusion...” “This experience made me change my perspective on respecting religion and beliefs...”	34.938	6.02	student, decis, separ, belief, religi
Topic 5	Gender Equality and Team Dynamics	“There is an inconsiderate and unequal view of a woman's abilities just because she is a woman...” “The society [should] advocate for social equality without discrimination of sex and gender...”	34.212	5.77	equal, dr, manag, sister, role
Topic 6	Institutional and Interpersonal Perspectives on Gender	“The two-day course in sex and gender medicine was for me a small taste of the turbulent world of reality in our small country, which is at the heart of a clash between East and West, between religion and liberalism, between boundless freedom and compulsive conservatism...” “It also connects to Israeli reality, particularly the case of Alice Miller, who applied to become a pilot and the President of Israel, who was himself a former pilot, responded to her by saying “Just as women were meant to knit socks, flying planes is the domain of men, and one cannot change the order of the world”...”	32.122	5.09	subject, sex, realiti, world, biolog
Topic 7	Psychological and Emotional Dimensions of Gender and Health	“I connected with my partner's feelings; we both felt the disdain from the doctor and were very hurt by him...” “In general, the issue of treating a patient's body with sensitivity in front of us as medical professionals is something that is very important to me, for example, not long ago, when a woman about my age in my community called me because of chest pain that required an EKG...”	30.317	4.53	partner, topic, pain, question, long
Topic 8	Gender and Decision-Making in Medicine	“During the treatment of the incident, which took place at the entrance to her home, the driver on my team, who is a paramedic by medical authority, lifted the woman's shirt to look for any other areas of rash besides what we saw...” “This incident stayed with me even later, on the next shifts I worked, and I literally promised myself that next time I would not let something like this happen when I was the team leader, and that even if I had unpleasantness in front of another team member, it was better than allowing something like this to happen...”	29.472	4.28	friend, hurt, team, bodi, high
Topic 9	Perceived Gender Bias in Clinical and Research Settings	“Meanwhile, Dr. ***'s presentation on the higher prevalence of autoimmune diseases in women and the lack of gender-oriented research in this field was eye-opening...” “Interpreting these lectures together, I see a pattern of systemic bias in health care and medical research that has long neglected women's specific needs and experiences...”	27.908	3.84	symptom, research, level, bias, underestim
Topic 10	Legal and Ethical Dimensions of Gendered Health Care	“After I got home, I searched for more information on Google and found that the law grants the woman the almost exclusive right to decide [whether to terminate or continue a pregnancy], while men, on the other hand, are not entitled to decide the issue except in very specific cases...” “The comparison to Iran, a country known for its restrictive laws toward women, expressed her feelings of oppression and coercion...”	25.898	3.31	convers, side, pregnanc, right, law

### Subanalysis by Ethnicity

The LSA of reflections from Jewish medical students (Table 5) revealed that the dominant theme, topic 1, accounted for 28.06% of the variance and reinforced the central, cross-cutting theme of Gendered Patient-Doctor Interactions, which consistently emerged as the core axis across all subgroups. Other key full-sample themes—such as gender-specific diseases, transgender health care, gender differences in pharmacology, gender and decision-making, and psychosocial dimensions—were retained in both lexical structure and conceptual scope. However, 2 new themes emerged in the Jewish-only analysis. Topic 5 suggested a discourse on population-level gendered conditions, possibly linking epidemiological thinking to gendered illness patterns, while topic 10 revealed a narrative centered on early socialization, gendered expectation formation, and informal educational settings.

The LSA of reflections from Arabic medical students (Table 6) explained 92.2% of the total variance, indicating a semantically rich and cohesive thematic structure.

Topic 1, which accounted for 35.95% of the total variance (eigenvalue=58.017), reaffirmed the central theme of Gendered Patient-Doctor Interactions. Topic 2 (13.31% variance) was likewise retained. Topic 3 represented a new theme, characterized by culturally specific terms that suggested heightened sensitivity to modesty, gendered exposure, and familial expectations in clinical contexts. Similarly, topic 5 introduced a novel discourse on paternal authority and structural discrimination, with terms pointing to familial and systemic influences on gender roles and health care access—elements particularly salient in Arabic sociocultural settings. Two other themes were reoriented from their original full-sample conceptualizations. Topic 4 captured procedural aspects of care and implicitly gendered experiences during physical examination and consultation. Topic 7 aligned with the previously defined theme of gendered communication but introduced a spatial and emotional safety dimension, with cultural reframing. Additional new themes were identified in topics 9 and 10. Topic 9 pointed to concerns around reproductive health and perceived vulnerability. Finally, topic 10 captured emotional burden and coping dynamics.

**Table 5:** Labeled topics identified through latent semantic analysis of student reflections following participation in a gender medicine course in the Jewish-only sample.

Topic	Label	Representative quotes	Eigenvalue	% Variability	Top words
Topic 1	Gendered Patient-Doctor Interactions	“I feel that as a professional expanding my knowledge in the field of medicine in general and in ECGs specifically, I underestimated the patient due to a lack of knowledge and the routine of daily life, the treating doctor did as well, as did her husband based on his understanding...” “I think and feel that as a professional and a future doctor we must listen to our patients...”	109.760	28.06	gender, patient, feel, women, doctor
Topic 2	Gender-Specific Diseases and Health Concerns	“I was surprised, because I thought that precisely because they have little breast tissue, the diagnosis would be earlier, but most of the time they do not check for changes in the breast at all...” “During the weight loss process, my brother had fatty tissue on his chest that bothered him and looked like female breasts...”	51.474	6.17	breast, cancer, partner, didn, hurt
Topic 3	Gender and Decision-Making in Medicine	“It is clear to me that a woman's individual rights and freedom in making decisions about her own body are fundamental principles that must be protected...” “It is important to recognize that the weight of the final decisions lies with the woman, but also to be sensitive to the needs and feelings of men in these situations...”	43.140	4.34	student, opinion, sister, decis, separ
Topic 4	Health Care Access for Transgender Individuals	“This was the first time I had interviewed a transgender person...” “I chose this topic because I believe it is a complex and important issue in the field of sex and gender-aware medicine. The complexity of treating transgender patients concerns not only medical aspects, but also behavioral and psychological ones, and I think it is very important to raise awareness of the issue, create a more supportive and respectful medical environment, and improve the quality of care and life of patients who have undergone this procedure...”	41.514	4.01	base, transgend, servic, unit, train
Topic 5	Gender and Public Health Framing of Conditions	“We are required to learn about the needs of [all] populations, regardless of religion, race, gender, or age, while providing comfort and a pleasant experience to every patient...” “Everyone was shocked, as there isn’t enough awareness of these conditions and the numbers, especially in this population!...”	39.559	3.65	condit, popul, month, arriv, week
Topic 6	Gender Differences in Pharmacology	“In a lecture, I learned about a drug that has a stronger effect on women, to the point that the dose needed is half that needed by men...” “It is clear that women, even if you take into account their body weight, often receive drugs in doses that are not suitable for them...”	35.990	3.02	lectur, drug, dose, menstrual, communiti
Topic 7	Gender Equality and Team Dynamics	“I always taught my paramedic trainees and colleagues along the way to listen to the patient, not to diminish their value or their feelings, to rule out and take things seriously-it’s life!...” “A specific event that I remember was a shift in which I was the team leader and worked with another male paramedic from the Arab sector...”	35.558	2.95	woman, team, incid, leader, paramed
Topic 8	Institutional and Interpersonal Perspectives on Gender	“Although my mother and friends told me a little about the subject [i.e., menstruation], they never went into depth, so I didn't exactly understand what it was about until it happened to me...” “Recently, at a regular gathering among friends, I was present at a conversation, on the subject of discrimination against men in unwanted pregnancies, a topic that has come up in the past...”	33.649	2.64	subject, studi, friend, realiti, biolog
Topic 9	Psychological and Emotional Dimensions of Gender and Health	“This may have been caused by a lack of understanding of the emotional and mental effects of the syndrome...” “I assume that this was due to the emotional difficulties that the syndrome brings with it...”	31.049	2.25	social, save, syndrom, attack, mention
Topic 10	Gendered Youth, Expectations, and Identity Formation	“It prompted a deeper examination of how societal expectations around gender impact not only health care but also various aspects of daily life, from career choices to personal relationships...” “I think we have to find the power in the decisions we want to make as independent, and not as something that society expects us to do...”	29.704	2.06	girl, meet, expect, don’t, camp

**Table 6:** Labeled topics identified through latent semantic analysis of student reflections following participation in a gender medicine course in the Arabic-only sample.

Topic	Label	Representative quotes	Eigenvalue	% Variability	Top words
Topic 1	Gendered Patient-Doctor Interactions	“Her doctor referred her to a specialist at the hospital in order to begin the treatment steps as early as possible...” “I understood that there is a price for mistakes and lack of adequate response to gender issues as an important part of the treatment...”	58.017	35.95	gender, women, medic, treatment, import
Topic 2	Gender Equality and Team Dynamics	“This incident taught me a lot about myself: I learned how hard it is for me to skip things that go against my values and don't fit my worldview, I learned how much I care about the health of the people around me and how important it is for them to feel good...” “My values, based on human dignity and equality, pushed me to act in the girl's best interest...”	35.304	13.31	dr, equal, manag, sister, valu
Topic 3	Cultural Norms in Clinical Settings	“At the time, she felt embarrassed by his reaction in front of the rest of the family and was hurt, which led to an argument between the two of them...” “After a long conversation, I was able to understand from her that she was afraid and did not know how to break the news to her parents due to embarrassment...”	28.317	8.56	aunt, society, situation, surgery, embarrass
Topic 4	Gendered Clinical Encounters	“I suggested that she postpone this step until after her consultation with the specialist, and at the end of the conversation, she asked me to accompany her to the hospital on that day...” “In my eyes, this is where the hardest part of the treatment began, because one of the steps of the surgery involved breaking the hymen to reach the polyp located on the cervix...”	25.686	7.05	pain, exam, step, consult, result
Topic 5	Paternal Authority and Structural Discrimination	“She claimed that her father and brothers did not believe her and supported her father's wife; her mother, who lived in Rahat after a divorce, did not object to the father returning her daughter home...” “Personally, the event strengthened my understanding of the importance of supporting and assisting people in distress, especially women who experience violence and discrimination...”	24.217	6.26	felt, father, explain, driver, discrimin
Topic 6	Psychological and Emotional Dimensions of Gender and Health	“This experience reinforced my understanding that mental and physical health are interconnected, and that comprehensive care must address the whole person...” “During her hospitalization, we worked to understand her experiences, her special needs as a new mother, and the impact of her mental state on her functioning as a mother...”	22.917	5.61	dose, mental, impact, clinic, provid
Topic 7	Gendered Communication and Assertion in Health Care Contexts	“The new doctor's approach was completely different, she addressed not only the physical aspects of the pain but also its emotional and social effects...” “I tried to talk to him sensitively at that moment because it was a family matter...”	20.968	4.70	emot, address, sens, room, start
Topic 8	Intergenerational and Familial Perspectives on Gendered Health	“The case ended with a compromise, in which the girl would move in with her mother...” “Following what happened, I think back to my own distant childhood and remember my mother, who is now a retired teacher and her education for me and my sister, who were the youngest children and how she educated us for equality, that whatever is fair for my sister is also fair for me...”	19.533	4.08	mother, disease, topic, face, factor
Topic 9	Reproductive Health and Feelings of Vulnerability	“First, I realized how much a lack of listening on the part of medical professionals can lead to a feeling of helplessness and loneliness in the patient...” “I chose to present this topic because it is an extremely important topic in psychiatry and medicine in general [i.e., postpartum depression], as it requires a special understanding of the physiological and emotional changes that women go through after childbirth...”	18.314	3.58	birth, touch, question, helpless, correct
Topic 10	Psychosocial Support and Gendered Distress	“I learned to appreciate the importance of social and psychological support and develop a more inclusive and understanding approach towards people in complex situations...” “This includes recognizing the emotional effects of illness on a patient’s life and understanding the importance of emotional support alongside medical care...”	16.941	3.07	support, girl, complex, distress, act

## Discussion

### Principal Findings

The findings of this study highlight the complex and multidimensional ways in which first-year medical students in Israel engage with the concept of gender in both educational and clinical contexts. LSA of student reflections revealed a dominant recurring theme—Gendered Patient-Doctor Interactions—across all analyses. This convergence aligns with prior scholarship underscoring the critical role of gender in shaping clinical communication, empathy, and trust [23]. The centrality of this theme suggests that even early exposure to gender medicine can prompt students to critically reflect on the influence of gender on therapeutic relationships and clinical outcomes [24,25]. In line with the World Health Organization’s assertion that gender is a fundamental social determinant of health [5], students in this study demonstrated an emerging awareness of how gender shapes illness narratives, health care access, and diagnostic pathways. Their reflections also echoed the “gender mainstreaming” approach promoted in international medical education policy, which advocates for integrating gender considerations at all levels of health care delivery and education [5,26,27]. Notably, themes related to transgender health care, gender-specific diseases, and pharmacological differences paralleled the growing literature that underscores the need to dismantle androcentric biases in medical curricula [28,29]. These insights suggest that when given structured opportunities for reflection, learners are capable of recognizing and critically interrogating structural inequities embedded within biomedical discourse and practice.

Rather than merely acknowledging the existence of inequities, participants were challenged to examine critically the structural foundations of medical knowledge production, clinical practice, and professional formation. Many expressed dismay at the historical exclusion of female bodies from clinical research and the persistent privileging of male physiology as the normative reference point. As one student succinctly articulated: “We were presented with the historical neglect of female subjects in clinical trials in various fields, and an approach that perpetuated biased medical treatments that favor male physiology as the default standard.” This statement reflected not only an awareness of bias but also a structural critique of the biomedical canon, echoing feminist analyses of knowledge systems that render women invisible or anomalous within clinical reasoning. Importantly, the student’s reference to “perpetuated biased medical treatments” underscored an understanding that these are not merely vestiges of the past but ongoing epistemic and clinical injustices. The course further encouraged students to reevaluate their professional identity and the ethics of care through a gender-responsive lens. Many participants described how the training reshaped their conception of good medical practice—shifting from a mechanistic, one-size-fits-all model to a more nuanced, context-sensitive, and individualized approach. One student reflected: “I learned about the importance of listening to patients and the need to treat them as individuals, and not just as representatives of a particular gender.” This shift marks a clear departure from biomedical reductionism, highlighting the importance of relational ethics and embodied listening—practices central to feminist clinical pedagogy. Importantly, the course also created space for critical emotional and identity work, particularly among male students. While some initially reacted with defensiveness or discomfort, perceiving gender-sensitive discourse as accusatory or polarizing, dialogical engagement and pedagogical scaffolding helped transform these affective responses into opportunities for deeper, transformative learning. As one male student candidly recounted: “At the beginning of the ‘Sex and Gender-Aware Medicine’ course, I felt, as a man, uncomfortable and even indirectly attacked...However, in small group exercises, the learning experience changed. The instructors helped me understand that the goal is not to blame, but to train us to be better doctors.” This reflection illustrates how pedagogical designs grounded in openness and dialogic learning can transmute resistance into productive introspection. The student’s movement from a sense of personal indictment toward professional growth signals a successful pedagogical intervention—one that shifts affective orientations while fostering ethical self-positioning. Crucially, the course did not stop at generating cognitive insights; it also catalyzed concrete commitments to behavioral change. Several students described actionable strategies for integrating sex- and gender-aware practices into their future patient encounters. One such example reads: “Following the course, I intend to ask more focused questions about the medical history of my patients, taking gender differences into account. I also intend to continue to stay up-to-date with research and literature in the field of gender medicine, in order to provide my patients with the best possible care.” Here, we witness the internalization of gender mainstreaming not as an abstract principle but as a guiding framework for everyday clinical decision-making and professional formation. Some students even extended their reflections beyond the medical *curriculum*, engaging with the broader sociocultural implications of gendered behavior and ethics. One student, for example, recounted a personal experience in which a pregnant woman was ignored on public transport, using this incident as a lens to interrogate gendered expectations, moral responsibility, and social awareness. While not strictly clinical, such reflections highlight the pervasiveness of gendered norms and underscore the importance of cultivating ethical responsiveness that transcends disciplinary boundaries.

The gender-stratified analysis revealed meaningful differences in both the conceptual emphasis and emotional tone of student reflections. Female students more frequently highlighted relational and affective dimensions of gender, including intergenerational caregiving roles, informal consultation, and communicative dynamics in health care. These findings align with prior research indicating that female medical students often adopt a relational epistemology and are more inclined to reflect on their positionality and ethical responsibilities in clinical practice [30]. By contrast, male students tended to engage more with the biomedical, legal, and structural dimensions of gender, raising issues such as diagnostic bias and reproductive rights. This pattern reflects what prior literature has described as a more abstract and normative framing of social issues among male learners [30].

Moreover, this study reveals that students construct divergent understandings of gender medicine through identity lenses deeply shaped by religion, ethnicity, ideology, and sociopolitical context. One particularly compelling reflection came from a student who described the course as exposing “the tumultuous reality in our small country, which is caught in the middle of a clash between East and West, between religion and liberalism.” The student reflected on the tension between their conservative upbringing and the realities of contemporary clinical practice, asking: “Is there room for the religious beliefs upon which my upbringing is based, or do I need to re-examine or abandon my personal beliefs and open up to secularism and liberalism?” These questions encapsulate the internal negotiation learners face when confronted with dissonant ideas that challenge their foundational schemas. Similarly, ethnic subgroup analyses highlighted the intersection of gender and cultural identity. Reflections from Jewish students revealed a tendency to extrapolate personal experiences to broader public health framings, often invoking themes such as epidemiological patterns and gendered health behaviors. This ability to connect micro-level experiences with macro-level perspectives aligns with what Cruess et al [31] describe as the development of the physician’s “professional identity.” Meanwhile, reflections from Arabic students revealed a deeper engagement with culturally embedded norms surrounding modesty, familial authority, and structural barriers to health care access—patterns consistent with scholarship on the intersection of gender, religion, and medicine in collectivist cultures [32]. Notably, these students also voiced affectively charged concerns related to reproductive health, vulnerability, and emotional burden, underscoring the cultural and emotional labor involved in reconciling conflicting expectations within clinical spaces [33,34]. Taken together, these reflections—shaped by diverse positionalities—illustrate how pedagogical encounters with gender can elicit complex and, at times, conflicting epistemic trajectories.

Overall, this study adds to the growing body of scholarship calling for the integration of gender and cultural competence into medical education. The themes identified suggest that structured gender curricula, when paired with reflective pedagogy and systematic evaluation, can cultivate critical consciousness and social responsiveness in future physicians [35].

Reflection—especially when systematically analyzed through computational methods—can serve as both a pedagogical and evaluative tool, enabling the assessment of not only cognitive but also affective and ethical dimensions of learning [36].

### Implications of Findings

The findings of this study carry important implications for the design and implementation of gender medicine curricula, particularly in culturally and identity-diverse contexts. The subgroup differences observed—for instance, female students’ greater emphasis on relational and affective dimensions, or Arabic students’ focus on culturally embedded norms and structural constraints—demonstrate that learners do not uniformly assimilate gender medicine content. Rather than passively absorbing knowledge, students actively interpret and reframe new information through the lens of prior experiences, sociocultural background, and identity. This process reflects constructivist learning theory, which emphasizes that learning is active, situated, and coconstructed. Gender and ethnic identity often function as interpretive filters, amplifying certain issues while diminishing others. Such identity-based perspectives can enrich the learning environment by contributing diverse viewpoints, yet they may also generate tensions when personal or cultural values conflict with curricular content. From a pedagogical perspective, these findings underscore the importance of embedding structured opportunities for students to articulate and critically examine their own perspectives. Dialogical learning spaces, culturally responsive facilitation, and peer-to-peer engagement can help learners navigate potentially dissonant concepts and integrate them into their emerging professional identity. Such strategies may bridge the gap between abstract knowledge of gender medicine and its practical application across diverse clinical contexts. At the institutional level, embedding gender medicine within an explicitly intersectional framework ensures that the interwoven effects of gender, ethnicity, religion, and other social determinants are systematically addressed. This approach can strengthen both cultural competence and the ability to provide equitable, patient-centered care to heterogeneous populations.

### Strengths and Limitations

Nonetheless, while the results are promising, caution is warranted. The interpretability of LSA findings is contingent on robust preprocessing, linguistic clarity within the student corpus, and careful topic labeling. Although this study benefited from certain strengths, including stratification by gender and ethnicity, the limited sample size precluded a comprehensive intersectional analysis. Furthermore, the reflections were collected at a single time point and situated within a specific institutional and sociocultural context, which may constrain the transferability of findings. Longitudinal studies are needed to assess whether these conceptual gains translate into sustained behavioral change and improved clinical competence in gender-sensitive care. In addition, comparative research across medical schools and national contexts could illuminate how curricular design, institutional culture, and sociopolitical environments shape the uptake and integration of gender medicine.

### Conclusions

This study underscores the importance of embedding gender-sensitive content within medical education and demonstrates the utility of LSA as an analytic tool for examining how learners engage with complex sociomedical themes. The findings highlight the need for an intersectional, contextually attuned approach to curriculum design that actively considers students’ diverse identities and lived experiences. By fostering reflexivity, empathy, and critical literacy, gender medicine education holds the potential to shape future physicians who are better equipped to provide equitable, inclusive, and socially responsive health care.

## Abbreviations

DIEP: Describe, Interpret, Evaluate, and Plan
LSA: latent semantic analysis
